# Effects of retinoic acid on compensatory lung growth

**DOI:** 10.1186/1749-8090-3-37

**Published:** 2008-06-30

**Authors:** Sami Karapolat, Aydin Sanli, Ahmet Onen, Unal Acikel, Oya Sivrikoz

**Affiliations:** 1 Department of Thoracic Surgery, Dokuz Eylul Medical School, Izmir, Turkey; 2 Department of Pathology, Sifa Hospital, Izmir, Turkey

## Abstract

**Background:**

We investigated the effect of Retinoic acid in the growth of contralateral lung after pneumonectomy.

**Methods:**

Twentyone adult male Wistar albino rats from the same colony were used. They were divided into three groups (Group A, B and C). Group A undergone only left posterolateral thoracotomy. In Group B and C, the rats were subjected to left posterolateral thoracotomy and left pneumonectomy. In Group C, rats were given intraperitoneal Retinoic acid during the operation and continued to be given everyday postoperatively. Rats were sacrificed on the 10^th ^day and their total body, right lung weights and right lung volumes were measured.

**Results:**

The volume and weight indices of the lung were found to be higher in Group C. In histopathological examination, there was a reduction in the mean number of alveoli in Group B and C. A significant rise in the mean dimension and average wall thickness of the alveolar structure were determined in Group C.

**Conclusion:**

Retinoic acid contributes to the compensatory growth of the residual lung tissue.

## Introduction

One of the most serious problems that may develop after lung resection is the situation that the existent remaining lung tissue becomes insufficient and can not provide enough oxygenization for the body. In most of these cases, no symptom is seen during rest, but with efforts, complaints like shortness of breath, palpitation and quick tiring due to insufficient oxygenization occur. This situation restricts the functional capacity, lengthens the time to return to daily activities and worsens the postoperative quality of life [1,2]. The hospitalization and loss of labour also increase.

There occurs some growth in the residual lung tissue physiologically postoperatively. The volume, weight, collagen content, protein and cell size increase in the residual lung [3]. However, its not being in desirable degrees creates problems. This is especially important for the patients having limited respiratory reserve in the respiratory function tests performed preoperatively. Thus, providing a dimensional increase in the residual lung tissue contributes to the improvement of respiratory functions. For this purpose, lots of pharmacologic agents have been used but, a few of them have given positive results. Retinoic acid (RA) is a product of Vitamin A metabolism and promotes cell growth. It is a powerful signaling molecule controlling cell proliferation via specialized transcription factors, the nuclear RA receptors. It is one of the important factors controlling the growth of foetal lung and is necessary for the development of the lung. RA also affects the development of trachea and bronchopulmonary structures besides lung tissue [4,5]. The use of RA in lobectomy or pneumonectomy cases postoperatively increases the lung growth and may create a positive effect on its functions. If RA might be effective, the growth in the residual lung tissue would rise the quality of life in the applied lung resection cases and would shorten the period to return back to normal life.

The purpose of this experimental study is to investigate whether there will be a growth in the volume and weight of the residual lung and an increase in the functional capacity in rats that undergone pneumonectomy, with RA treatment administered exogenously. If this becomes true, an important additional respiratory reserve contribution can be achieved in cases that undergone lobectomy and pneumonectomy, especially in those with limited respiratory reserve with the improvement of new treatment strategies. It may be possible to perform lung resection such as pneumonectomy easily in patients accepted as to be inoperable due to limited respiratory capacity as well.

## Materials and methods

Twentyone adult male Wistar albino rats with an average of 220–260 g body weight from the same colony were used. The purposes of using rats are their easy availability, safety and the high ratio of repeating the experiment [6]. The rats were cared for in accordance with the Guide for the Care and Use of Laboratory Animals. They were kept at 21° to 23°C, with controlled humidity, and a dark-light cycle of 12 to 12 h. Food and water were available ad libitum. The experimental protocol was approved by Dokuz Eylul University, School of Medicine, Animal Care and Use Committee.

Rats were divided into three groups randomly (Group A, B, and C). 1- Group A: Sham (n = 7), 2- Group B: Control (n = 7), 3- Group C: RA (n = 7). All rats were anesthetized by administering Ketamine (100 mg/kg/intraperitoneal) and Xylazine (5 mg/kg/intraperitoneal), their body weights were measured, and after tracheostomy, they were intubated with 16G catheter. Rats were given ventilator support using room atmosphere in volume control mode throughout operation. Ninety per minute respiration count and 15–20 ml/kg minute volume were provided during ventilation (Hugo sacs, rodent ventilator, Germany). Because mediastinal pleura is not complete, and is quite weak in rats. Unless a positive pressure ventilation is achieved, pleura is sutured and both lungs collapse even when only one pleura is opened [7].

In operation, rats were put into right posterolateral position; the site was shaved and disinfected by using povidone iodine. Group A has undergone only left posterolateral thoracotomy and the layers were closed properly after bleeding control.

Left posterolateral thoracotomy was applied to the rats in Group B and C, and left pneumonectomy was performed after ligation of hilus done with 4-0 silk suture. Then, bleeding and air leakage controls were done and the thoracotomy was closed properly.

When the spontaneous respiration was achieved, rats were extubated, tracheostomy was closed and they were kept in different cages. No drug treatment was applied to Group A and B. In Group C, rats were given intraperitoneal RA (0.5 microgram/g) during operation and the same dosage was continued to be given everyday postoperatively (Retinoic Acid Empirical Formula (Hill Notation) C20 H28 02, Formula weight: 300.44 Cas Number: 302-79-4, R-2625-100 mg, 98% (HPLC), powder, Sigma, USA). In the first 24 hours rats were given Buprenorphine 0.03 mg/kg every 8 hours subcutaneously. They were given food and water as much as they wanted. Wound infection has occurred in one rat (in Group B) and this rat was excluded from the experiment protocol. Rats were sacrificed at the end of 10^th ^day by administering lethal dose of Ketamine intraperitoneally. Then, their total body weight and right lung weight were measured. In order not to be affected by the tissue water, right lung weight was taken after lung tissue was removed and dried at room temperature over 3 hours.

Right lung volume was calculated according to the method defined by Scherle [8]. At this point, after placing and fixing a catheter to the right lung bronchi related with trachea, lung was inflated until it became well expanded and the volume was calculated. In order to prevent volume faults that can occur, right lung volume measurement was taken after each lung tissue had been expanded at maximum. The volume (ml) and weight (g) of the lung was compared with the body weight prior to sacrifice and the indices of lung weight and volume were found.

Lung Volume Index (LVI) = Lung volume/Total body weight (ml/kg)

Lung Weight Index (LWI) = Lung weight/Total body weight (g/kg)

The results were evaluated statistically. In order to evaluate the difference among experiment groups, Wilcoxon Signed Ranks Test and Kruskal Wallis Test were used. Statistically p > 0.05 was accepted as insignificant, and p < 0.05 as significant in these tests.

The lung specimens were fixed in 10% formalin, processed for paraffin embedding and then sectioned at 5 μm. Hematoxylin-Eosin stained sections were used to evaluate histopathological findings by light microscopy.

## Results

The weights of the rats were measured in grams at the initial stage of the experiment. When the experiment was finished, the rats were weighed once more. The body weight differences for each subject were calculated and their values were found as percentage. In Group A, B, and C; there are weight increases of 1.9%, 2.0% and 3.4% as the mean values, respectively. As it is seen, there is some weight increase in all groups at the end of the experiment. The most increase is observed in RA group.

**Figure 1:**
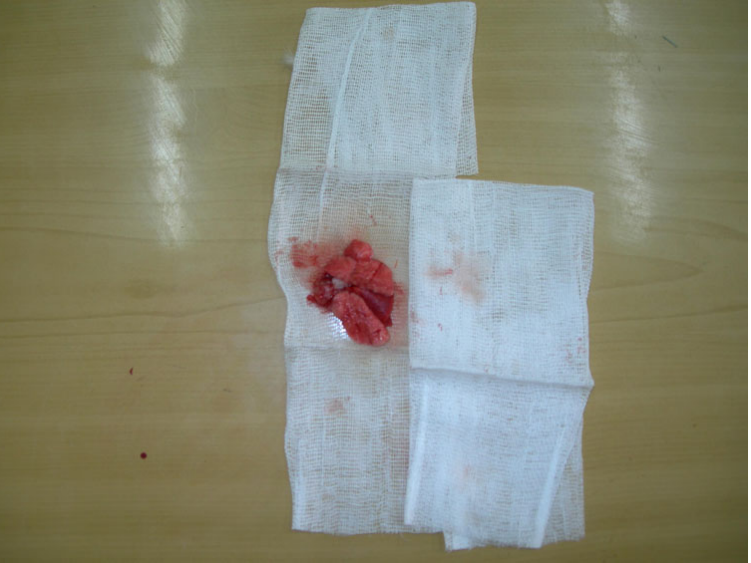
At the end of the experiment, right lung tissue of rats was removed. It was seen extend and lobulate position.

At the end of the experiment, the right lungs of rats were removed (Fig 1). The right lung volumes of the rats were measured in millilitre. Right lung volumes were between 2.8–3.4 ml (mean 3 ml) in Group A, 3.3–4.2 ml (mean 3.7 ml) in Group B, and 3.9–4.8 (mean 4.3 ml) in Group C.

Right lung volumes of Group B compared to Group A, and Group C to group A and B were found to be more.

**Figure 2:**
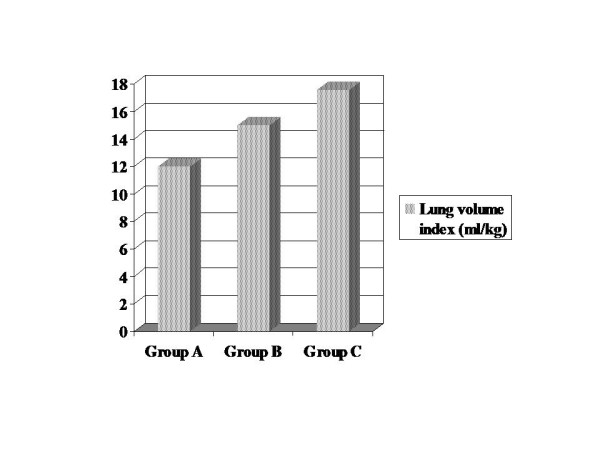
At the end of the experiment, lung volume indices of all groups were compared. It was found higher in Group C. There was statistically significant difference in Group B and C compared to Group A (p < 0.05). Also, a statistically significant difference was found between Group B and C (p < 0.05).

Lung volume indices were determined for each group. Lung volume indices were between 10.8–13.4 ml/kg in Group A (mean 12.1), 13.3–17.5 ml/kg in Group B (mean 15.0), and 15.2–19.6 ml/kg in Group C (mean 17.6). Lung volume indices were found higher in Group B compared to Group A, and in Group C compared to Group A and B (Fig 2).

At the final stage of the experiment, right lung weights of the rats were measured in grams. They were between 2.40–2.94 g in Group A (mean 2.68 g), 2.70–3.31 g in Group B (mean 2.99), and 3.44–3.78 g in Group C (mean 3.59).

Right lung weight was found to be more in Group B compared to Group A, and in Group C compared to Groups A and B.

**Figure 3:**
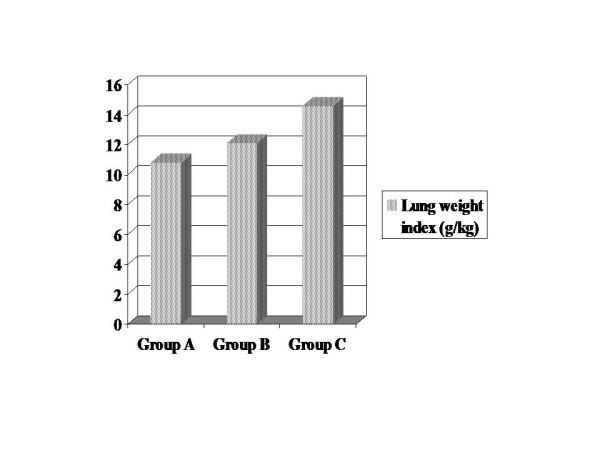
At the end of the experiment, lung weight indices of all groups were compared. It was found higher in Group C. There was statistically significant difference was found between Group A and Group B (p < 0.05), and significant difference between Group A and Group C (p < 0.05). Also, there was a statistically significant difference between Group B and Group C (p < 0.05).

Lung weight indices were determined for each group. Lung weight indices in Group A were between 9.2–12.0 g/kg (mean 10.8), 11.0–13.5 g/kg in Group B (mean 12.1), and 13.8–15.8 g/kg in Group C (mean 14.6). As it is seen, lung weight indices increased in Group B compared to Group A, and in Group C compared to Groups A and B (Fig 3).

There was no statistically significant difference between the body weights of Groups when the initial and final days of the experiment were compared (p > 0.05).

When the right lung volumes among the groups were compared at the end of the experiment, there was statistically significant difference between Group A-B (z = -2.4, p < 0.05), and Group A-C (z = -2.4, p < 0.05) and a statistically significant difference between Group B-C (z = -2.2, p < 0.05).

When the lung volume indices were compared, there was statistically significant difference in Group B and C compared to Group A (z = -2.4, p < 0.05). A statistically significant difference was found between Group B and C (z = -2.2, p < 0.05).

The right lung weights were compared and there found to be a statistically significant difference between Group A and B (z = -2.2, p < 0.05), and statistically significant differences between Group A-C and Group B-C (z = -2.4, p < 0.05).

When the lung weight indices were compared, a statistically significant difference was found between Group A and Group B (z = -2.4, p < 0.05), and significant difference between Group A and Group C (z = -2.6, p < 0.05). There was a statistically significant difference between Group B and Group C (z = -2.4, p < 0.05).

In all these statistical evaluations, Wilcoxon Signed Ranks Test, one of nonparametrical tests, was used.

The values of body weight on the 1^st ^and 10^th ^days and the body weight increase and total body weight percentage values at the end of the experiment were subjected to mutual statistical evaluation and no significant difference was found.

Right lung volume measurements were found to be statistically very significant in respect to volume among Groups A, B and C (x^2 ^= 15.67, sv = 2, p < 0.001). Lung volume indices were found to be statistically very significant among Groups A, B and C (x^2 ^= 15.96, sv = 2, p < 0.001). Right lung weight measurements were found to be statistically very significant from the point of weight among Groups A, B and C (x^2 ^= 15.39, sv = 2, p < 0.001). Also, lung weight indices were statistically very significant among Groups A, B and C (x^2 ^= 14.64, sv = 2, p < 0.05) (Table 1).

**Table 1:** All of groups were compared by statistically (Kruskal Wallis Test).

	**Body ** **weight** **(1^**st **^day)**	**Body ** **weight** **(10^**th **^day)**	**Increase ** **on body** **weight** **(%)**	**Total** ** body ** **weight** ** (g)**	**Right** **lung ** ** volume ** **(ml)**	**Lung** ** volume ** **index** **(ml/kg)**	**Right** ** lung ** **weight ** **(g)**	**Lung** ** weight ** **index** **(g/kg)**
**p**	0.313	0.509	0.713	0.485	0.000	0.000	0.000	0.001

**p: **p value (Significance was accepted as p < 0.05.)

These evaluations were made by means of a nonparametric test, Kruskal Wallis Test.

In the histopathological examination, the alveoli numbers in per 5 high-power fields of each histopathological preparation prepared separately for each subject were calculated and in the end, individual means of these values were found for each group. As a result, the mean number of alveoli in per 5 high-power fields was found to be 24.0 ± 1.3 in Group A, 20.6 ± 1.1 in Group B, and 16.8 ± 1.1 in Group C. A statistically significant difference was found between Group A and Group B (p < 0.05), and significant difference between Group A and Group C (p < 0.05). Also, there was a statistically significant difference between Group B and Group C (p < 0.05).

The mean alveolus dimension was found by measuring 5 different alveoli dimension in each histopathological preparation prepared separately for each subject and then by calculating the mean of these values for each group. The mean alveolus dimension was found as 1.76 ± 0.04 μ in Group A, 2.92 ± 0.02 μ in Group B, and 4.06 ± 0.03 μ in Group C. A statistically significant difference was found between Group A and Group B (p < 0.05), and significant difference between Group A and Group C (p < 0.05). Also, there was a statistically significant difference between Group B and Group C (p < 0.05).

**Figure 4:**
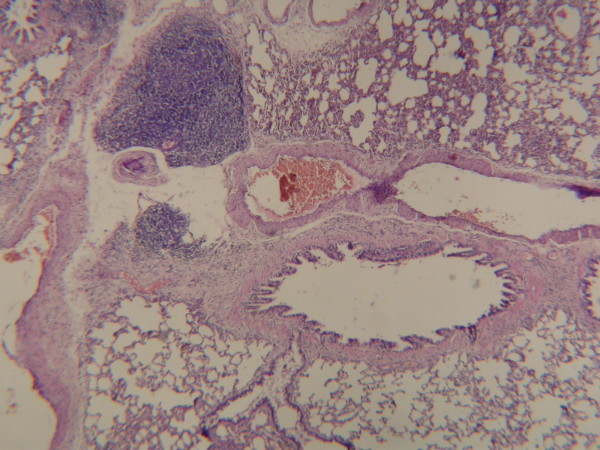
In Group A, normally lung tissue was observed on histopathological examination (Hematoxylin-Eosin, original magnification ×200).

**Figure 5:**
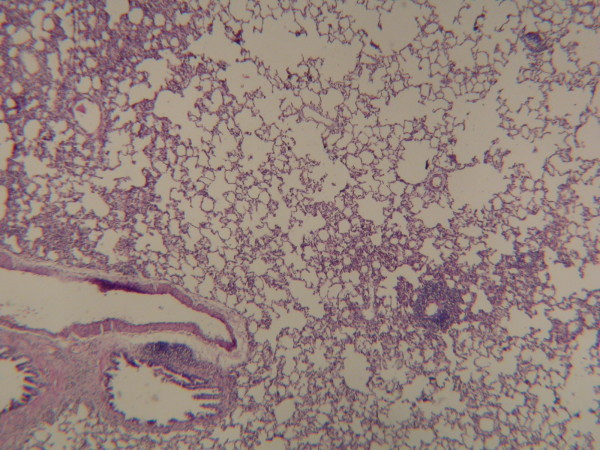
In Group B, an increase in the dimensions of alveoli on lung tissue was observed (Hematoxylin-Eosin, original magnification ×200).

**Figure 6:**
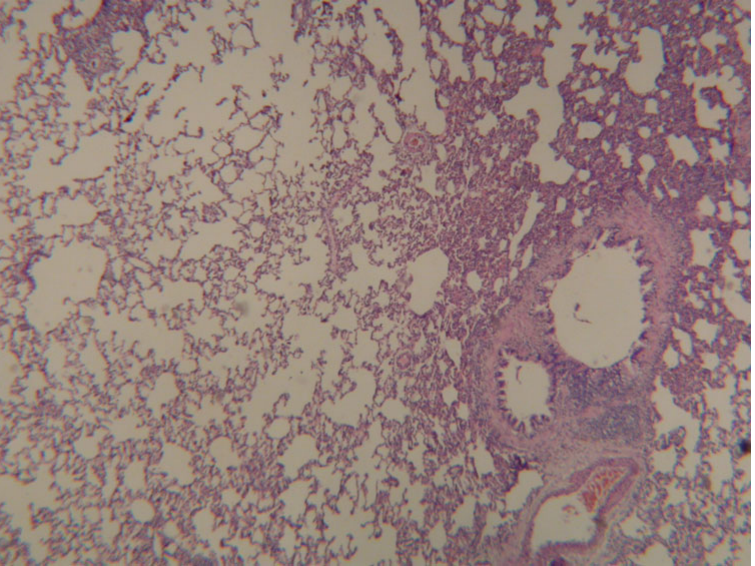
In Group C, a significant increase in the dimensions and thickening in the walls of alveoli was observed (Hematoxylin-Eosin, original magnification ×200).

The mean alveolar wall thickness was found by measuring the wall thickness of 5 different alveoli in each histopathological preparation prepared separately for each subject, and then by calculating the means of these values for each group. The mean alveolar wall thickness was 0.68 ± 0.02 μ in Group A, 1.0 ± 0.04 μ in Group B, and 1.2 ± 0.04 μ in Group C (Fig 4, 5, 6). A statistically significant difference was found between Group A and Group B (p < 0.05), and significant difference between Group A and Group C (p < 0.05). However, there was not a statistically significant difference between Group B and Group C (p > 0.05).

## Discussion

The main problem in applied lung resection patients, who indeed have a limited capacity of respiration, is the respiration functioning efficiency problems that can be encountered after the operation. This situation is not so common because preoperatively the patients are subjected to respiratory function tests by spirometry, their arterial blood gas is examined and if needed, their quantitative ventilation-perfusion scintigraphies are taken. Surgical process is performed according to these results if patients are suitable. In addition, the remaining lung tissue is also not healthy in these patients and most of which cilliary functions are out of order, compliance is decreased, and have damage due to chronic infection [9]. In conclusion, residual lung may be in difficulty in meeting the sufficient respiration during an effort postoperatively. Such situation decreases the functional capacity and lengthens the time to return to daily activities [10].

Such problems developing postoperatively are usually seen in pneumonectomy patients. But, also lobectomy sometimes leads to reduction of lung function whether patients complain of dyspnea on effort or not. In addition, compensatory lung growth is not a response only peculiar to pneumonectomy patients. Regenaration of the lungs also occur in patients who had undergone whether anatomical surgical procedure such as lobectomy and segmentectomy or nonanatomical surgical procedure like wedge resection. Rannels et al. reported that this regeneration response occurs via adrenal steroids and growth hormone [9]. Postlobectomy lung growth is a more rapid and restorative process. On the contrary, the space occurring in hemithorax due to compensatory lung growth fills in time and no air space remains in most of the patients [11]. In addition, Kaza et al. have reported that, compensatory lung growth after lobectomy occurs with cellular hyperplasia in early period and with cellular hypertrophy in late period [12,13]. In this study, compensatory lung growth after pneumonectomy is just a model to evaluate the possible role of retinoic acid in regeneration of the lung.

Postpneumonectomic compensation develops in two phases. In the initial phase of active cellular growth, septal thickening, lengthening in bronchioalveolar tract and limited functional adaptation occur together. In the late phase, septal remodeling restores the normal alveolar structure, and this phase occurs together with great functional increase. When the compensatory lung growth is complete; alveolar and capillary cells and the surface area of blood gas barrier protect their position mostly [14,15].

Gaining a dimensional increase in residual lung tissue contributes to the improvement of respiratory functions. A number of pharmacologic agents have been used for this purpose. One of these is RA [16,17]. Cell culture proliferation of Type 2 pneumonocytes can be induced by means of RA treatment. Besides, RA increases the production of surfactant and alveolization. It also increases the collagen on the walls of respiratory tract. In addition, it plays an important role in the alveolar epithelial cell proliferation. It increases the mechanical tightness in lung tissue and induces the progressive compensatory growth of new alveolar tissue. RA increases the endothelial cells and capillary growth selectively. Yet, it does not affect the growth of the other alveolar cell types [16].

RA has been used in lots of studies because of these known effects. One of these studies is randomized, double-blind, feasibility study carried out by Roth et al. In this study, 148 subjects with chronic obstructive pulmonary disease and whose primary component was emphysema were divided into three groups and received low, high doses of RA and placebo, respectively. This treatment lasted for six months. In this study, retinoid-related side effects were found to be common but mild. In conclusion, with the administration of RA, there were no definitive clinical benefits in the patients. However, time and dose-dependent changes in diffusing capacity of the lung for carbon monoxide and health related quality of life were observed in RA group. The authors stated that, these results supported the possibility of biological activity by means of the administration of RA and for this reason there is a need for more investigation [18].

In this study, the compensatory growth occurring in the contralateral lung by administering RA to the rats after left pneumonectomy was controlled. It was administered to the Group C as 0.5 microgram/g for 10 days. Lung volume indices and lung weight indices were used for the evaluation of the results. These are the values that can be calculated quantitatively and that can give information about lung growth.

There was some weight increase in the group to which RA was administered. This may be explained by the knowledge that, Vitamin A generally plays a part in the cellular growth of all tissues. However, when the weight gain is compared with the other groups, it is seen that it is not significant statistically (p > 0.05).

When the rats were sacrificed, right lung tissues were examined macroscopically both when those were in thorax and those were removed. It was observed that right lung tissue showed herniation to the left hemithorax much more in Group C, and partially in Group B and C anteriolaterally, and more from posterior prevertebral space. Those posterior herniations seen more commonly after left pneumonectomy are more in Group C shows a proportionally increased herniation with right lung growth.

Right lung tissues in each of the three groups were removed by turning from the right main bronchus and separating the surrounding fibrotic adhesions. A very careful dissection should be performed in all these steps as paranchyme destruction may affect the lung weight, cause air leakage and thus lead to wrong volume measurements.

Total volume of the lungs of rats is generally between 5.0–5.5 ml although it shows changes from the point of age and sex. Right lung volumes in Group B showed increase compared to Group A, and in Group C when compared to Groups A and B. Average right lung volume to which RA administered reached above 4 ml. This situation verifies the alveolar expansion and the size increase due to this. The reason for choosing the 10^th ^day as the measurement time is that the upregulation of molecular mediators in growth period occurs in this duration. It was reported previously that the right lung growth reached plateau between 10^th ^and 21^th ^days [19]. Therefore, a 50% or more volume increase was determined with a ten-day-treatment protocol. This is a little more above than expected. However, how long the RA treatment would continue and when the maximum response would be were not known thoroughly, a ten-day-treatment program was applied. Some more studies should be carried out on RA in respect to duration and response axis depending on this time.

Lung volume index is a useful parameter that can be calculated quantitatively. Since there is the body weight in denominator part, it is affected negatively by the weight increase occurred in rats. Yet, the weight increase and the volume growth will lead to some increase in lung tissue of the rats. Thus, the changes in these two ratios will balance each other, and a reliable value will be obtained as a result. In our study, the lung volume indices in Group B increased compared to Group A, and in Group C when compared to Groups A and B. The mean increase in index of Group C is about 45–50%. This shows that, when the most weight increase in Group C is taken into account, the lung volume increase in the group administered RA is much more than the others. The amount in the volume increase also affects the index. As the lung volume index determines the volume amount of lungs according to the body weight, it can give information only about the lung volume amount, and it is not a functional indicator.

Mean rat dry lung weight is between 5–9 g though it shows differences from the point of weight, age and sex. When the right lung weights are evaluated, an increase about 20% in Group B, and about 40% in Group C were observed. From this point of view it is understood that the compensatory lung growth caused some increase in the lung weight. The administration of RA doubled this increase. Of course, as the weight increase in Group C is more than the other groups, the lung weight to occur would be affected from this situation. In other words, the final lung weight increase should be accepted a little lower than the value for Group C. However, this situation should not overshadow the existing significant difference.

In the evaluation of the lung tissue growth, the lung weight index value can be calculated quantitatively and similarly to the volume index. It can be obtained dividing the provided lung weight by total body weight. As in the volume index, since the body weight is in the denominator, it is negatively affected by the weight increase occurred in rats. However, since the increase occurred in lung weight shows a parallelism to total weight gain, lung weight index may remain fixed without being affected from these. The lung weight indices calculated at the end of the experiment increased in Group B compared to Group A, and in Group C compared to other groups. The increase in this index in Group C is about 40–45% as an average. That is, lung weight increase is more in the rats of administered RA. Since the lung weight index determines the weight amount of lungs according to the body weight, it can give information only about lung size, and it is not a functional indicator. On the other hand, the information reached shows that RA leads to a real growth in the lung tissue, and that the alveolar surface on the expands as its tissue weight has increased, and thus these result in an increase in the total effective lung volume. This shows that the lung growth by administration of RA after pneumonectomy is increased significantly as a result of the evidences of the increase in these two indices.

In the investigation of the experiments carried out on the postpneumonectomic lung growth, the studies of Kaza et al. attract attention [19]. Kaza et al. indicated the positive effects of RA and claimed that it provides a significant functional contribution. The same authors used epidermal growth factor and keratinocyte growth factor for this purpose, and again reached successful results [12,20].

Epidermal growth factor normally plays an important role in prenatal and postnatal lung development. In postresectional lung growth, on the other hand, this can be explained by the increase in the amount of growth factor receptors of residual lung tissue. On the other side, keratinocyte growth factor plays a role in pneumocyte proliferation and lung development. Postresectional lung tissue also induces new alveolar formation.

Sakamaki et al. used hepatocyte growth factor for the same purpose [21]. Hepatocyte growth factor has mitogenic and morphogenic actions on lung epithelial cells. As a result of the study, it was found that hepatocyte growth factor increased the deoxyribonucleic acid synthesis in lung epithelial cells in rats undergone pneumonectomy and that it was a pulmotrophic factor.

Desai et al. investigated RA from the point of its effect on lung morphogenesis in mouse foregut cultures and reported that RA affected the mesodermal proliferation and induced fibroblast growth factor expression [22]. Nevertheless, Sakurai et al. observed in their experimental study that compensatory lung growth was angiogenesis- dependent and was accelerated by vascular endothelial growth factor but not affected by fibroblast growth factor [23].

Finally, all these growth factors can be effective on compensatory lung growth. They achieve these effects usually by providing the upregulation of their own receptors in lung tissue. Among these, the lung growth most resembling to the result obtained by RA was achieved by using keratinocyte growth factor and epidermal growth factor. RA was preferred in this study due to its easy availability, cheapness, easy usage and low side effect incidence.

In our study, histopathological results were evaluated in addition to lung weight index and lung volume index. There was no pathological finding in Group A (Fig 4). An increase in the dimensions of alveoli in the right lung tissue of Group B was observed (Fig 5). We also observed a significant increase in the dimension of the alveoli in Group C. Besides, a thickening in the walls of alveoli was determined (Fig 6).

In the histopathological examination, the mean alveoli number in per 5 high-power fields was found to be the most in Group A, and this value got less in Group B and Group C. If the mean alveolar dimension values are to be compared among groups, there was nearly a 50% increase in Group B compared with Group A, and this increase outran the value of Group A twice as much as Group C.

When the mean alveoli thickening values were compared among groups, there was a 30% increase in Group B compared with Group A, and a 70% increase in Group C compared with Group A.

In conclusion, we think that the fall in the mean alveoli number in Group B and C results from the alveolar hypertrophy developing in these groups. The increases in the dimension and wall thickness of the alveoli in Group B and C lead to decrease in the number of alveoli for per unit field.

The mean alveolus dimension and the mean alveolar wall thickness were found increased in Group B compared with sham group and this supports the postpneumonectomic compensatory lung growth expected to occur to some degree normally. These increases were observed to be more in Group C. This aspect partially supports the positive effects of RA on compensatory lung growth. However, the mean alveolar wall thickness of Group B and C was compared statistically, and there was not a statistically significant difference between Group B and Group C (p > 0.05). This situation may support that, the increase of the mean alveolar wall thickness on pneumonectomy normally occurs, and RA has not much affected this value.

The initial stimulus of the compensatory lung growth to occur during postpneumonectomic period is the mechanical tightening in the residual lung tissue. This tightening will increase the wall tightening of the cells in the existing tissue and this will possibly lead to expansion in volume. The compliance in the expanded lung tissues will increase and try to fill the area by moving towards the pneumonectomic space. These areas physiological changes are observed as routine. Yet, the compensation occurred differs from an individual to another, and its amount and effectiveness are different. In order to solve this problem, RA can be used in the light of the information we obtained. RA administration in the early postoperative period increases the alveolar capillary amount and the size of endothelial cells and causes the formation of new capillaries. As a result, the celluler growth increases and lung functions improve [24]. Septum thickens is an important ratio. The increase occurred in the amount of capillaries can be explained by the increase in the vascular endothelial growth factor. Administration of RA increases both the upregulation of its own receptors and the amount of growth receptors in the residual lung tissue. Epidermal growth factor may play a role in this process. In this way, there occurs a restoration in the levels of all protein, Deoxyribonucleic acid, collagen and elastin in the residual lung due to this complex receptor-mediator relation that took place [15,25].

It is controversial how long the RA treatment will last. In some studies, it is indicated that there is a ten-day-treatment period, and if more, that there occurs a stagnation period in lung growth [19]. An evident growth takes place at the end of the first week. The remodeling of the lung takes place in 21 days in normal people. As a result, it is thought that during postpneumonectomic early period, a few weeks treatment is enough. The usage of RA for more than one month is controversial. The dosage to be used is also an important subject. A suitable dosage not having side effects and showing physiological effects should be determined. Finally, RA is deposited in fat, and high dosage for a long time may lead to toxicity.

## Conclusion

Protecting the body and survival, and securing a postresectional compensatory lung growth, can be accelerated by administering RA exogenously. Starting the treatment in the early period will secure to obtain satisfactory results. Lung growth due to RA administration should be carried out to increase the comfort and quality of life of these patients. Thus, the hospitalization period and drug usage may decrease, the resistance of the patient to environmental factors and microorganisms will increase, annual doctor visit number may lessen, and the necessary additional strength to continue daily activities of the patients having limited lung function capacities can be provided. It may not be possible to compensate completely the function of lung tissue by this treatment. However, due to the positive effects of RA mentioned earlier, growing the residual lung tissue may cause not to feel the functional loss or to feel it much less. When the improvement in living comfort of the patients is taken into account, this treatment protocol can be a choice to be tried in necessary situations. Nevertheless, all these views should be arranged in terms of the results to be obtained from clinical Phase 1 and Phase 2 studies that RA is administered on human.

## Conflict of interest statement

The authors declare that they have no competing interests.

## Authors' contributions

SK and AS carried out the surgical procedures, and the design of the study. AO participated in the design of the study, and performed the statistical analysis. UA conceived of the study, and participated in its coordination. OS carried out the histopathological examination. All authors read and approved the final manuscript.
